# A Large-Scale Genome-Wide Association Study of Epistasis Effects of Production Traits and Daughter Pregnancy Rate in U.S. Holstein Cattle

**DOI:** 10.3390/genes12071089

**Published:** 2021-07-18

**Authors:** Dzianis Prakapenka, Zuoxiang Liang, Jicai Jiang, Li Ma, Yang Da

**Affiliations:** 1 Department of Animal Science, University of Minnesota, Saint Paul, MN 55108, USA; praka032@umn.edu (D.P.); zliang@umn.edu (Z.L.); 2 Department of Animal Science, North Carolina State University, Raleigh, NC 27695, USA; jicai_jiang@ncsu.edu; 3 Department of Animal and Avian Sciences, University of Maryland, College Park, MD 20742, USA; lima@umd.edu

**Keywords:** epistasis, GWAS, milk production, fertility

## Abstract

Epistasis is widely considered important, but epistasis studies lag those of SNP effects. Genome-wide association study (GWAS) using 76,109 SNPs and 294,079 first-lactation Holstein cows was conducted for testing pairwise epistasis effects of five production traits and three fertility traits: milk yield (MY), fat yield (FY), protein yield (PY), fat percentage (FPC), protein percentage (PPC), and daughter pregnancy rate (DPR). Among the top 50,000 pairwise epistasis effects of each trait, the five production traits had large chromosome regions with intra-chromosome epistasis. The percentage of inter-chromosome epistasis effects was 1.9% for FPC, 1.6% for PPC, 10.6% for MY, 29.9% for FY, 39.3% for PY, and 84.2% for DPR. Of the 50,000 epistasis effects, the number of significant effects with log10(1/p) ≥ 12 was 50,000 for FPC and PPC, and 10,508, 4763, 4637 and 1 for MY, FY, PY and DPR, respectively, and A × A effects were the most frequent epistasis effects for all traits. Majority of the inter-chromosome epistasis effects of FPC across all chromosomes involved a Chr14 region containing *DGAT1*, indicating a potential regulatory role of this Chr14 region affecting all chromosomes for FPC. The epistasis results provided new understanding about the genetic mechanism underlying quantitative traits in Holstein cattle.

## 1. Introduction

Epistasis effects are widely considered important [1,2,3,4], but the number of reported epistasis effects is far behind the number of single-point effects [2,4,5]. Genome-wide association study (GWAS) in several dairy cattle breeds have reported many single-SNP effects on dairy traits [6,7,8,9,10,11,12,13,14,15,16,17,18,19,20]. However, epistasis studies in dairy cattle were limited to candidate gene tests [21,22,23] or had a small sample size [24]. The U.S. Holstein cattle have uniquely large sample sizes [25,26] and provide an opportunity to identify epistasis effects associated with dairy traits using GWAS. The purpose of this study was to identify pairwise epistasis effects associated with five dairy production traits and one fertility trait using the same Holstein cattle population that we previously used for a large-scale GWAS to detect SNP additive and dominance effects [19]. For each SNP pair, we tested four types of epistasis effects, additive × additive, additive × dominance, dominance × additive, and dominance × dominance, and investigated the intra- and inter-chromosome epistasis effects.

## 2. Materials and Methods

### 2.1. Holstein Populations and Genotyping Data

The sample for GWAS analysis contained 294,079 first lactation Holstein cows with phenotypic observations for five milk production traits (milk, fat and protein yields, and fat and protein percentages), and one fertility trait (daughter pregnancy rate). Daughter pregnancy rate is the percentage of cows that become pregnant during each 21-d period [26]. The number of phenotypic observations ranged from 294,079 for milk yield to 245,214 for daughter pregnancy rate. The 294,079 cows had two sets of SNP genotypes, the 80 K with original and imputed 76,109 SNPs with minor allele frequency of 0.05 and the 60 K with original and imputed 60,671 SNPs. Both the 80 K and 60 K SNPs were based on the same set of SNP chips previously described [19]. The 80 K is used in the current U.S. Holstein genomic evaluation, whereas the 60 K was used for the U.S. Holstein genomic evaluation when we reported SNP additive and dominance effects [19]. Results in this article were based on the 80 K except three inter-chromosome epistasis figures for fat percentage that used the 60 K results because the 60 K had more inter-chromosome epistasis effects for fat percentage than the 80 K. SNP positions were based on the ARS-UCD1.2 bovine genome assembly.

### 2.2. GWAS Analysis

The GWAS analysis used the method of an approximate generalized least squares (AGLS) analysis [19,27]. The AGLS method combines the least squares (LS) tests implemented by EPISNP1mpi [28,29] with the estimated breeding values from routine genetic evaluation using the entire U.S. Holstein population as a covariable. The statistical model was:

(1) $$y-Z\tilde{a}=µI+X_{g}g+Za+e=Xb+Za+e$$

where **y** = column vector of phenotypic deviation after removing fixed nongenetic effects such as heard-year-season (termed as ‘yield deviation’ for any trait) using a standard procedure for the CDCB/USDA genetic and genomic evaluation; $\tilde{a}$ = column vector of 2(PTA), PTA = predicted transmission ability from routine genetic evaluation, µ = common mean; **I** = identity matrix; **g** = column vector of pairwise genotypic values; **X**_g_ = model matrix of **g**; **b** = (μ, **g**′)′, **X** = (**I**, **X**_g_); **a** = column vector of additive polygenic values; **Z** = model matrix of **a** = identity matrix if each individual has one observation; **e** = random residuals. The first and second moments of Equation (1) are E(**y**) = **Xb**, and var(**y**) = **V** = **ZGZ**ʹ + **R** = $\sigma_{a}^{2}$**ZAZ**ʹ + $\sigma_{e}^{2}$**I**, where $\sigma_{a}^{2}$ = additive variance, **A** = additive relationship matrix, and $\sigma_{e}^{2}$ = residual variance. Equation (1) achieves the benefit of sample stratification correction from mixed models without the computing difficulty of inverting **V** or **A**. Four types of epistasis effects were tested: additive × additive (A × A), additive × dominance (A × D), dominance × additive (D × A), and dominance × dominance (D × D). The significance tests used the t-tests of the epistasis contrasts of the estimated SNP genotypic values [10,30], and the t-statistic was calculated as:

(2) $$t_{j}=\frac{{|L}_{j}|}{\sqrt{{\mathrm{var}(L}_{j})}}=\frac{\left|s_{j}^{}\hat{g}\right|}{v\sqrt{s_{j}^{}{\left(X_{}^{\prime }\;X_{}\right)}_{\mathrm{gg}}^{-}s_{j}^{\prime }}},\;\;j\;=\;A\times A,\;A\times D,\;D\times A,\;D\times D$$

where $L_{j}$ = epistasis contrast, $\sqrt{{\mathrm{var}(L}_{j})}$ = standard deviation of the additive or dominance contrast, $v^{2}=\left(y-X\hat{b}\;)\;\prime \;(y-X\hat{b}\right)/(n-k)$ = estimated residual variance; $\hat{g}$ = column vector of the AGLS estimates of the pairwise SNP genotypic effects ${\left(X_{}^{\prime }X\right)}_{\mathrm{gg}}^{-}$ = submatrix of ${\left(X_{}^{\prime }X_{}\right)}_{}^{-}$ corresponding to $\hat{g}$. A limitation in EPISNP1mpi causes a p value less than 10^−308^ to be printed as ‘0’. For such p values, empirical p values as a power function of the observed t-values were calculated using the empirical formula of $p\;=\;0{.2416(t}^{1.9713})$ [19]. For 76,109 SNPs and eight traits, the total number of pairwise tests was 2,896,251,886 × 4 × 8 = 92,680,060,352. The threshold p value with the Bonferroni correction for 0.05 genome-wide false positives was p = 5.39(10^−13^), and a pairwise epistasis effect was declared significant if log10(1/p) > 12.26 ≈ 12. The Manhattan plots of observed log10(1/p) values for each trait were made using SNPEVG2 version 3.2 [31] for a global view of statistical significance, and the circular plots for global views of intra- and inter-chromosome epistasis effects were created using CIRCOS version 0.69-9 [32,33]. The 29 autosomes and the X chromosome had 1225–4121 SNPs per chromosome. These SNPs had 104,203,650 intra-chromosome SNP pairs and 2,791,287,201 inter-chromosome SNP pairs, or, 3.6% of all SNP pairs were intra-chromosome and 96.4% were inter-chromosome. The ratio of (A × A):(A × D + D × A):(D × D) tests was 1:2:1. These percentages and ratios were used for comparison with the observed percentages and ratios.

## 3. Results

### 3.1. Overview of Epistasis Effects

**Figure 1:**
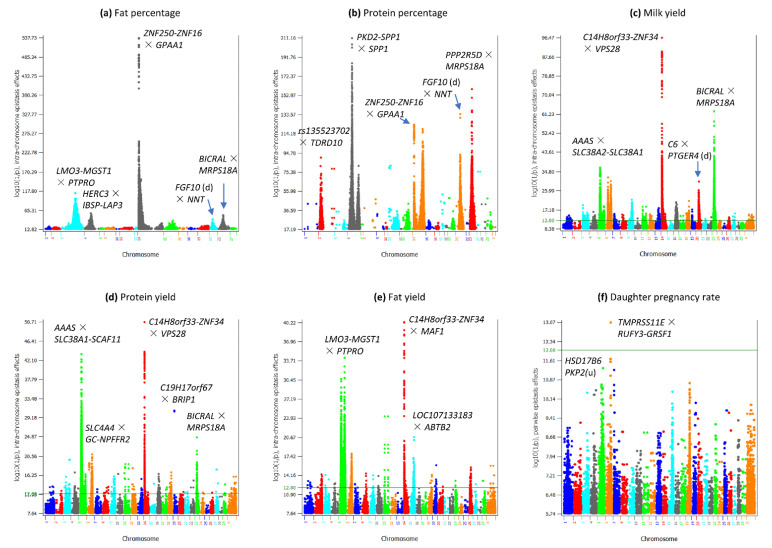
Manhattan plots of statistical significance of intra-chromosome epistasis effects for five milk production traits and daughter pregnancy rate. (**a**): Fat percentage. (**b**): Protein percentage. (**c**): Milk yield. (**d**): Protein yield. (**e**): Fat yield. (**f**): Daughter pregnancy rate.

**Figure 2:**
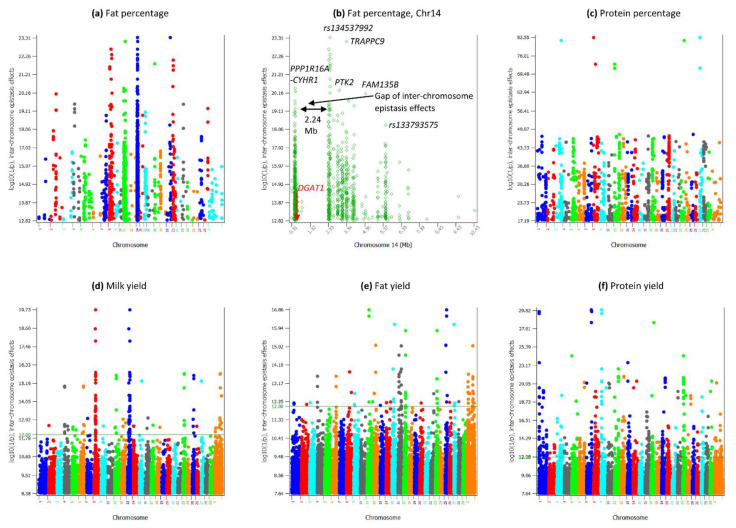
Manhattan plots of statistical significance of inter-chromosome epistasis effects for milk production traits. (**a**): Fat percentage. (**b**): Inter-chromosome epistasis effects of the 0.3-10.5 Mb region of Chr14 for fat percentage. (**c**): Protein percentage. (**d**): Milk yield. (**e**): Fat yield. (**f**): Protein yield.

The pairwise epistasis analysis focused on the top 50,000 pairs of epistasis effects for each trait. All 50,000 SNP pairs of fat and protein percentages had significant epistasis effects; milk yield had 10,485 (21.5%), fat yield had 4746, (10.4%), protein yield had 4629 (15.4%) significant epistasis effects, and daughter pregnancy rate only had one significant epistasis effect (Table 1). The six traits had sharp contrasts in percentages of intra- and inter-chromosome epistasis effects and in the distribution of A × A, A × D, D × A and D × D effects among the top 50,000 SNP pairs. Fat percentage (98.1%) and protein percentages (98.4%) had the highest frequencies of intra-chromosome epistasis effects, followed by milk yield (89.4%), fat yield (70.1%), protein yield (61.7%), and daughter pregnancy rate (15.8%) (Table 1). The A × A effects were the most frequent effects (Table 2). A major difference between daughter pregnancy rate and the five production traits was that inter-chromosome A × A effects were the most frequent A × A effects, 52.9% of the 50,000 effects for this trait. In contrast, protein yield that had the highest frequency of inter-chromosome epistasis effects among the five production traits had 34.9% of inter-chromosome A × A effects (Table 2). A global view of statistical significance of the top 50,000 epistasis effects for each trait is shown in Figure 1 for intra-chromosome epistasis effects and in Figure 2 for inter-chromosome epistasis effects. Significant epistasis effects for the five production traits are shown in Figure S1 for intra-chromosome epistasis, and in Figure S2 for inter-chromosome epistasis. Selected tests results including locations and log10(1/p) values of significant epistasis effects are given in Table S1 for intra-chromosome epistasis effects, and Table S2 for inter-chromosome epistasis effects.

### 3.2. Intra-Chromosome Epistasis Effects of Fat Percentage

Five chromosomes had the most significant intra-chromosome epistasis effects for fat percentage, chromosomes 14, 5, 6, 23 and 20, and 13 chromosomes did not have significant intra-chromosome epistasis effects for fat percentage, chromosomes 1, 4, 7, 12, 17, 21, 22, 24, 25, 26, 28, 29 and X (Figure 1a). The five chromosomes with the highly significant epistasis effects also had highly significant SNP effects [19]. This trait had the highest frequency of A × A effects, 97.4% of the top 50,000 SNP pairs had A × A effects, and the second highest frequency of intra-chromosome effects, 98.1% of the top 50,000 pairs had intra-chromosome epistasis effects (Table 2).

**Figure 3:**
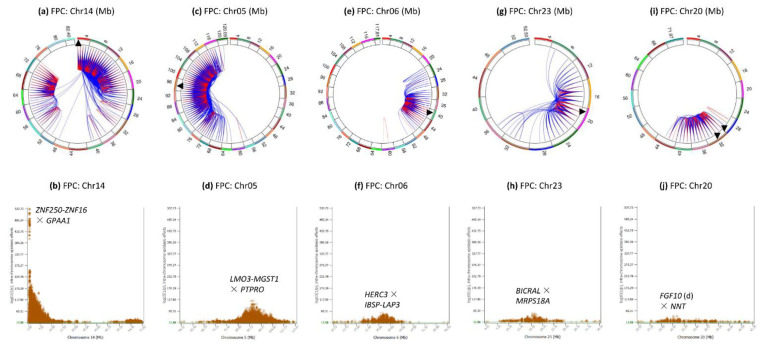
Chromosomes with most significant intra-chromosome epistasis effects for fat percentage. The top row shows the circular plots with red color indicating intra-4 Mb epistasis effect and blue color indicating inter-4 Mb epistasis effects; the bottom row shows the Manhattan plots of log10(1/p) values. The black arrow indicates the location of the peak epistasis effect of the chromosome. Gene name indicates the gene with a peak SNP epistasis effect, and ‘-’ indicates the SNP was between the two genes. FPC = fat percentage. (**a**): Intra-chromosome epistasis effects of Chr14 for fat percentage. (**b**): Statistical significance of intra-chromosome epistasis effects of Chr14 for fat percentage. (**c**): Intra-chromosome epistasis effects of Chr05 for fat percentage. (**d**): Statistical significance of intra-chromosome epistasis effects of Chr05 for fat percentage. (**e**): Intra-chromosome epistasis effects of Chr06 for fat percentage. (**f**): Statistical significance of intra-chromosome epistasis effects of Chr06 for fat percentage. (**g**): Intra-chromosome epistasis effects of Chr23 for fat percentage. (**h**): Statistical significance of intra-chromosome epistasis effects of Chr23 for fat percentage. (**i**): Intra-chromosome epistasis effects of Chr20 for fat percentage. (**j**): Statistical significance of intra-chromosome epistasis effects of Chr20 for fat percentage.

Chr14 had intra-chromosome epistasis effects in two regions: the 0.25–40 Mb region and the 52–74 Mb region (Figure 3a), but the latter was not among the most significant regions (Figure 1a and Figure 3b). These two regions had the largest number of intra-chromosome epistasis effects, 27,806 of the 49,046 pairs, or 56.7% of all intra-chromosome epistasis effects, but the 0.3–40 Mb region had the most significant and the largest number of effects. Of the top 82 most significant intra-chromosome epistasis effects, 76 pairs each was between two SNPs flanking *DGAT1*. The genes with such SNPs upstream of *DGAT1* included *ZNF250*, *ZNF16*, *C14H8orf33*, *ZNF34*, *CYHR1*, *VPS28*; the genes downstream of *DGAT1* included *GPAA1*, *MAF1*, *MROH1* and *PARP10*. The 2.86–3.77 Mb region with *PTK2*, *AGO2*, *CHRAC1*, *TRAPPC9* and *KCNK9* had the highest concentration of intra-chromosome epistasis effects, 10,963 pairs out of the 27,806 Chr14 SNP pairs, or 39.4% of the Chr14 intra-chromosome epistasis effects. The most significant intra-chromosome epistasis effect was between *rs110508680* (*ZNF250-ZNF16*) and *rs110929299* in *GPAA1*. The *DGAT1* gene was not heavily involved in intra-chromosome epistasis effect for any of the five milk production traits, with #2245 ranking and 120 epistasis effects for fat percentage, although *DGAT1* had the most significant SNP effects for all five milk production traits in the same dataset [19]. As to be described, *DGAT1* also was not heavily involved in inter-chromosome epistasis effects.

Chr05 had intra-chromosome epistasis effects in the 64–116 Mb region (Figure 3c), about half of the chromosome, with the second largest number of intra-chromosome epistasis effects, 17,057 epistasis effects, or 34.8% of all intra-chromosome epistasis effects. The most significant intra-chromosome epistasis effect of this chromosome was between *rs41602750* (*LMO3-MGST1*) and *rs136373712* in *PTPRO* (Figure 3d). We reported this region to have the most significant SNP effects of fat percentage among all chromosomes except Chr14 [19].

Chr06 had intra-chromosome epistasis effects mostly in the 32–44 Mb region (Figure 3e) with peak effect between *rs109552132* in *HERC3* and *rs109263339* (*IBSP-LAP3*) (Figure 3f). The 32–44 Mb region had significant SNP effects for all five production traits [19]. The top 100 pairs were located in the 30.15–36.70 region with *HERC3*, *IBSP*, *LAP3*, *LCORL*, *SPP1*, *MEPE*, *PKD2*, *CCSER1*, and *ABCG2*.

Chr23 had intra-chromosome epistasis effects in the 8–24 Mb region with peak with peak effects in the 16–17 Mb region (Figure 3g), and the most significant effect on this chromosome was between *rs109288796* in *BICRAL* and *rs109567499* in *MRPS18A* (Figure 3h). The Chr23 epistasis effects were mostly D × D, A × D and D × A effects, and most of the D × D effects involved genotype combinations with small frequencies of < 0.0001 (Table S1). These effects were in the same region where significant SNP dominance effects were detected [19].

Chr20 had intra-chromosome epistasis effects in the 25–43 Mb region (Figure 3i) with peak effect between *rs134342292* about 10 Kb downstream of *FGF10* and *rs41580285* in *NNT* (Figure 3j). The 25–43 Mb region contained the *GHR-C6-PRLR* region that had highly significant SNP effects for all five production traits [19].

### 3.3. Intra-Chromosome Epistasis Effects of Protein Percentage

Five chromosomes had the most significant intra-chromosome epistasis effects for protein percentage, chromosomes 14, 6, 23, 20 and 3; six chromosomes did not have such effects for protein percentage, chromosomes 2, 4, 21, 24, 25 and 26 (Figure 1b). The five chromosomes with the highly significant epistasis effects also had highly significant SNP effects [19]. This trait had the second highest frequency of A × A effects, 97.4% of the top 50,000 SNP pairs had A × A effects, and the second highest frequency of intra-chromosome effects, 98.4% of the top 50,000 pairs had intra-chromosome epistasis effects (Table 2).

**Figure 4:**
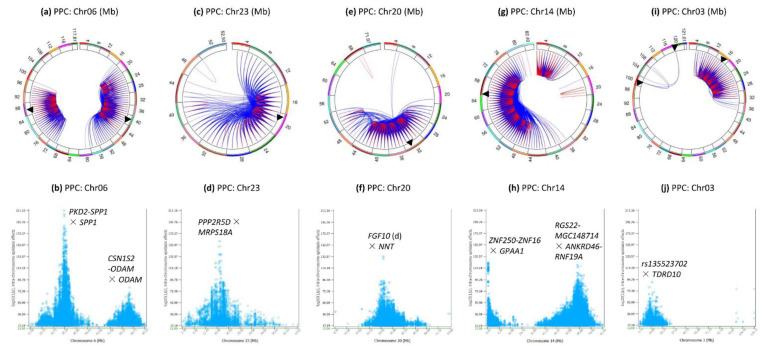
Chromosomes with most significant intra-chromosome epistasis effects for protein percentage. The top row shows the circular plots with red color indicating intra-4 Mb epistasis effect and blue color indicating inter-4 Mb epistasis effects; the bottom row shows the Manhattan plots of log10(1/p) values. The black arrow indicates the location of the peak epistasis effect of the chromosome. Gene name indicates the gene with a peak SNP epistasis effect, and ‘-’ indicates the SNP was between the two genes. PPC = protein percentage. (**a**): Intra-chromosome epistasis effects of Chr06 for protein percentage. (**b**): Statistical significance of intra-chromosome epistasis effects of Chr06 for protein percentage. (**c**): Intra-chromosome epistasis effects of Chr23 for protein percentage. (**d**): Statistical significance of intra-chromosome epistasis effects of Chr20 for protein percentage. (**e**): Intra-chromosome epistasis effects of Chr20 for protein percentage. (**f**): Statistical significance of intra-chromosome epistasis effects of Chr14 for protein percentage. (**g**): Intra-chromosome epistasis effects of Chr14 for protein percentage. (**h**): Statistical significance of intra-chromosome epistasis effects of Chr14 for protein percentage. (**i**): Intra-chromosome epistasis effects of Chr03 for protein percentage. (**j**): Statistical significance of intra-chromosome epistasis effects of Chr03 for protein percentage.

Chr06 had intra-chromosome epistasis effects in two regions: the 16–60 Mb region (approximately the same region with epistasis effects for fat percentage) and the 70–96 Mb region (Figure 4a). The most significant effects of all chromosomes were located in the 30.64–38.33 Mb region of Chr06 with *PKD2*, *SPP1*, *MEPE*, *IBSP*, *HERC3*, *ABCG2*; whereas the most significant effects within the 70–96 Mb region were located in 84.83–86.07 Mb with *CSN1S2*, *ODAM*, *CSN1S1*, *CSN2*, *LOC526979* (Figure 4b).

Chr23 had intra-chromosome epistasis effects in 12–24 Mb regions (Figure 4c), with peak effects in 13.85–18.61 Mb in or near *PPP2R5D*, *MRPS18A*, *BICRAL*, *FOXP4*, *SUPT3H*, *MOCS1*, *CCND3*, approximately the same region with significant epistasis and SNP dominance effects for fat percentage (Figure 4d). The Chr23 epistasis effects were mostly D × D, A × D and D × A effects, and most of the D × D effects involved genotype combinations with small frequencies < 0.0001. 

Chr20 had intra-chromosome epistasis effects in the 34–52 Mb region (Figure 4e) with peak effects in the 30.64–31.17 Mb of Chr20 involving a SNP 42 Kb downstream of *FGF10* and a SNP in *NNT* (Figure 4f). 

Chr14 had intra-chromosome epistasis effects in two regions, the 0.25–20 Mb and the 36–82.4 Mb regions (Figure 4g). Peak intra-chromosome epistasis effects were in the 0.25–0.81 Mb region involving SNPs in or near *ZNF250*, *ZNF16*, *GPAA1*, *MAF1*, *CYHR1*, *MROH1*, *VPS28*; in the 64.36–67.10 Mb region in or near *RGS22*, *MGC148714, VPS13B*, *OSR2*, and *CPQ* (Figure 4h).

Chr03 had regions had intra-chromosome epistasis effects mostly in the 4–30 Mb region (Figure 4i), with peak effects in the 15.9–16.0 Mb region in or near *TDRD10*, *KCNN3*, *ADAR*; between *rs135012389* at 98.6 Mb and *rs136744726* at 116.1 Mb in *LOC101904767* (Figure 4j).

### 3.4. Intra-Chromosome Epistasis Effects of Milk Yield

**Figure 5:**
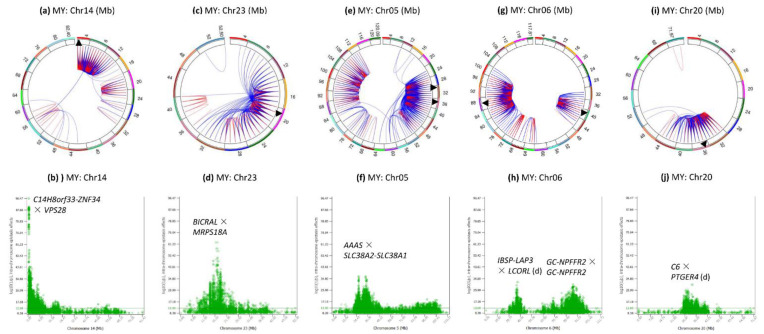
Chromosomes with most significant intra-chromosome epistasis effects for milk yield. The top row shows the circular plots with red color indicating intra-4 Mb epistasis effect and blue color indicating inter-4 Mb epistasis effects; the bottom row shows the Manhattan plots of log10(1/p) values. The black arrow indicates the location of the peak epistasis effect of the chromosome. Gene name indicates the gene with a peak SNP epistasis effect, and ‘-’ indicates the SNP was between the two genes. MY = milk yield. (**a**): Intra-chromosome epistasis effects of Chr14 for milk yield. (**b**): Statistical significance of intra-chromosome epistasis effects of Chr14 for milk yield. (**c**): Intra-chromosome epistasis effects of Chr23 for milk yield. (**d**): Statistical significance of intra-chromosome epistasis effects of Chr23 for milk yield. (**e**): Intra-chromosome epistasis effects of Chr05 for milk yield. (**f**): Statistical significance of intra-chromosome epistasis effects of Chr05 for milk yield. (**g**): Intra-chromosome epistasis effects of Chr06 for milk yield. (**h**): Statistical significance of intra-chromosome epistasis effects of Chr06 for milk yield. (**i**): Intra-chromosome epistasis effects of Chr20 for milk yield. (**j**): Statistical significance of intra-chromosome epistasis effects of Chr20 for milk yield.

Chromosomes 14, 5, 6, 23 and 20 had the most significant intra-chromosome epistasis effects (Figure 1c and Figure 5), and all the chromosome regions with peak epistasis effects also had peak SNP effects [19].

Chr14 had 2976 or 28.51% of the 10,440 intra-chromosome epistasis effects for milk yield mostly in the 0.25–12 Mb region (Figure 5a), with peak effect between *rs109752439* and *VPS28* (Figure 5b) that was also the peak effect for protein yield (Figure 1e). However, *DGAT1* that had the most significant SNP effects for all five production traits [19] only had five intra-chromosome epistasis effects with the best ranking of #6140 for milk yield and had no significant intra-chromosome epistasis effects for protein and fat yields.

Chr23 had intra-chromosome epistasis effects mostly in the 10–30 Mb region (Figure 5c), with peak D × D effects between two SNPs in *BICRAL* and *MRPS18A* in the 16.4–17.2 Mb region (Figure 5d). The 13.85–19.79 Mb region had a large number of D × D, D × A or A × D effects of milk and protein yields (Table S1) and was approximately the same region where significant SNP dominance effects were detected [19].

Chr05 had intra-chromosome epistasis effects mostly in the 24–50 and 84–112 Mb regions for milk yield (Figure 5e) with peak D × A effect between the dominance effect of a SNP in *AAAS* and the additive effect of a SNP between *SLC38A2* and *SLC38A1* (Figure 5f). In the 24–50 Mb region, majority of the epistasis effects were D × A, A × D and D × D. The chromosome region around the peak epistasis effect had highly significant dominance effects in or near *ATF7*, *AAAS*, *EIF4B* and *PLXNC1* [19].

Chr06 had two regions with intra-chromosome epistasis effects for the milk yield: the 30–46 Mb and the 62–102 Mb (Figure 5g), with peak effects between rs109263339 (*IBSP-LAP3*) and *rs110012183* downstream of *LCORL* in the first region, and between a SNP (*rs109452259*) flanked by *GC* and *NPFFR2* and multiple SNPs in *SLC4A4* in the second region (Figure 5h). The *SLC4A4- GC-NPFFR2* region had the most significant SNP effects on milk and protein yields outside Chr14 [19].

Chr20 had intra-chromosome epistasis effects in the 30–52 Mb region for milk yield (Figure 5i) with peak effect between a SNP in *C6* and a SNP in *PTGER4* (Figure 5j). The 25–43 Mb region contained the *GHR-C6-PRLR* region that had highly significant SNP effects for milk and protein yields [19].

### 3.5. Intra-Chromosome Epistasis Effects of Protein Yield

**Figure 6:**
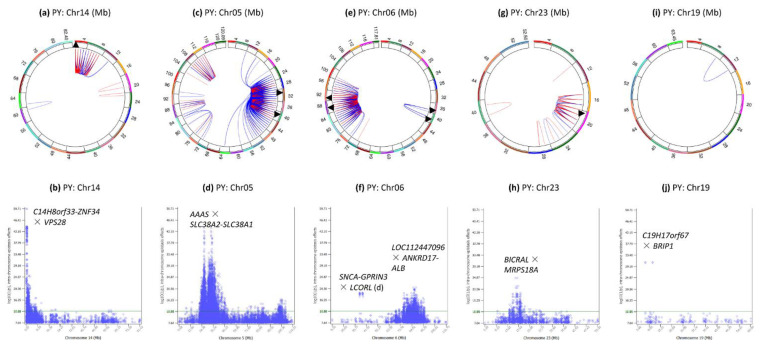
Chromosomes with most significant intra-chromosome epistasis effects for protein yield. The top row shows the circular plots with red color indicating intra-4 Mb epistasis effect and blue color indicating inter-4 Mb epistasis effects; the bottom row shows the Manhattan plots of log10(1/p) values. The black arrow indicates the location of the peak epistasis effect of the chromosome. Gene name indicates the gene with a peak SNP epistasis effect, and ‘-’ indicates the SNP was between the two genes. PY = protein yield. (**a**): Intra-chromosome epistasis effects of Chr14 for protein yield. (**b**): Statistical significance of intra-chromosome epistasis effects of Chr14 for protein yield. (**c**): Intra-chromosome epistasis effects of Chr05 for protein yield. (**d**): Statistical significance of intra-chromosome epistasis effects of Chr05 for protein yield. (**e**): Intra-chromosome epistasis effects of Chr06 for protein yield. (**f**): Statistical significance of intra-chromosome epistasis effects of Chr06 for protein yield. (**g**): Intra-chromosome epistasis effects of Chr23 for protein yield. (**h**): Statistical significance of intra-chromosome epistasis effects of Chr23 for protein yield. (**i**): Intra-chromosome epistasis effects of Chr19 for protein yield. (**j**): Statistical significance of intra-chromosome epistasis effects of Chr19 for protein yield.

Chromosomes 14, 5, 6, 23 and 20 had the most significant intra-chromosome epistasis effects (Figure 1d and Figure 6), and all the chromosome regions with peak epistasis effects also had peak SNP effects [19]. Chromosome 28 was the only chromosome without intra-chromosome epistasis effects with log10(1/p) ≥ 12 (Figure 1d).

Chr14 had intra-chromosome epistasis effects for protein yield mostly in the 0.25–7 Mb region (Figure 6a), with peak effect between *rs109752439* and *VPS28* (Figure 6b) that was also the peak effect for milk yield (Figure 5b).

Chr05 had intra-chromosome epistasis effects mostly in the 24–40 Mb for protein yield (Figure 6c) with peak D × A effect between the dominance effect of a SNP in *AAAS* and the additive effect of a SNP between *SLC38A2* and *SLC38A1* (Figure 6d). The same SNP pair also had peak D × A for milk yield. As for milk yield, majority of the epistasis effects in the 24–40 Mb region were D × A, A × D and D × D. Genes with highly significant intra-chromosome epistasis effects near the peak effects included *ATF7*, *AAAS*, *SLC38A1*, *SCAF11* and *ANO6*.

Chr06 had two regions with intra-chromosome epistasis effects for protein yield at 35.1–38.3 and 76–96 Mb (Figure 6e), with peak effects between *rs1095336909* (*SNCA*-*GPRIN3*) and *rs110718778* downstream of *LCORL* in the first region, and between *rs109452259* in *LOC112447096* and *rs135020479* flanked by *ANKRD17* and *ALB* (Figure 6f). A SNP (*rs109452259*) between *GC* and *NPFFR2* and five SNPs in *SLC4A4* had the #2–7 peak effects of the chromosome, noting that this region also had highly significant SNP effects for protein yield [19].

Chr23 had intra-chromosome epistasis effects mostly in the 14–20 Mb region (Figure 6g), with peak D × D effects between two SNPs in *BICRAL* and *MRPS18A* in the 16.4–17.2 Mb region (Figure 6h), the same SNP pair with peak D × D for milk yield (Figure 5d). The 13.85–19.79 Mb region had a large number of D × D, D × A or A × D effects of milk and protein yields (Table S1) and was approximately the same region where significant SNP dominance effects were detected [19].

Chr19 only had two pairs of highly significant intra-chromosome epistasis effect between *rs110881085* in *C19H17orf67* and two SNPs in *BRIP1* and *INTS2* (Figure 1d and Figure 6i,j).

### 3.6. Intra-Chromosome Epistasis Effects of Fat Yield

**Figure 7:**
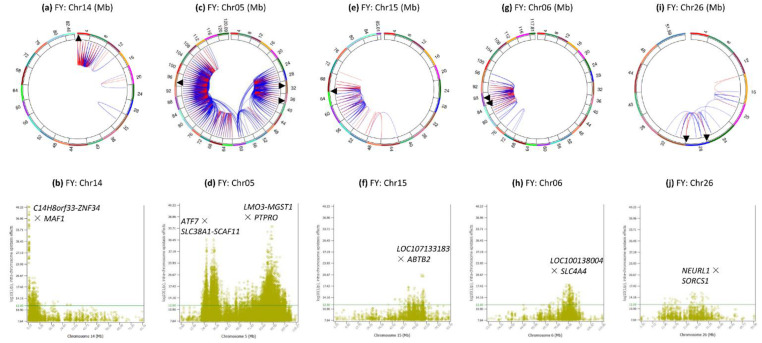
Chromosomes with most significant intra-chromosome epistasis effects for fat yield. The top row shows the circular plots with red color indicating intra-4 Mb epistasis effect and blue color indicating inter-4 Mb epistasis effects; the bottom row shows the Manhattan plots of log10(1/p) values. The black arrow indicates the location of the peak epistasis effect of the chromosome. Gene name indicates the gene with a peak SNP epistasis effect, and ‘-’ indicates the SNP was between the two genes. FY = fat yield. (**a**): Intra-chromosome epistasis effects of Chr14 for fat yield. (**b**): Statistical significance of intra-chromosome epistasis effects of Chr14 for fat yield. (**c**): Intra-chromosome epistasis effects of Chr05 for fat yield. (**d**): Statistical significance of intra-chromosome epistasis effects of Chr05 for fat yield. (**e**): Intra-chromosome epistasis effects of Chr15 for fat yield. (**f**): Statistical significance of intra-chromosome epistasis effects of Chr15 for fat yield. (**g**): Intra-chromosome epistasis effects of Chr06 for fat yield. (**h**): Statistical significance of intra-chromosome epistasis effects of Chr06 for fat yield. (**i**): Intra-chromosome epistasis effects of Chr26 for fat yield. (**j**): Statistical significance of intra-chromosome epistasis effects of Chr26 for fat yield.

Chromosomes 14, 5, 15, 6 and 26 had the most significant intra-chromosome epistasis effects (Figure 1e and Figure 7). Nine chromosomes did not have intra-chromosome epistasis effects with log10(1/p) ≥ 12, chromosomes 12, 13, 16, 17, 21, 22, 24, 25, and 28.

Chr14 had 259 intra-chromosome epistasis effects for fat yield mostly in the 0.24–8.11 Mb region (Figure 7a), with the most significant effects between *rs109752439* (*C14H8orf33-ZNF34*) and *MAF1* (Figure 7b) among all chromosomes. This 0.24–8.11 Mb region had 36 of the top 66 intra-chromosome epistasis effects and each of the 36 effects involved two SNPs on both sides of *DGAT1*, which did not have any significant intra-chromosome epistasis effect for fat yield.

Chr05 had 4111 or 86.31% of the 4763 intra-chromosome epistasis effects in the 22–112 Mb region that covered 93% of the chromosome (Figure 7c). The most significant effect was between *rs136373712* in *PTPRO* and *rs41602750* (*LMO3-MGST1*) (Figure 7d), and the 89.3–94.38 Mb region with *PTPRO*, *LMO3*, *MGST1*, *PDE3A*, *SLC15A5*, *DERA*, *PIK3C2G*, *RERGL* and *PLEKHA5* had the most significant A × A effects on this chromosome. The epistasis effects in the 26.5–44.9 region mostly involved dominance, A × D, D × A and D × D, with the most significant such effect being D × A between the dominance effect of *rs109675908* in *ATF7* and the additive effect of *rs42851612* (*SLC38A1-SCAF11*) (Figure 7d).

Chr15 had intra-chromosome epistasis effects mostly in the 52–66 Mb region (Figure 7e) with peak effect between *rs132656140* in *LOC107133183* and *rs29022897* in *ABTB2* in the 63.51–64.79 Mb region (Figure 7f).

Chr06 had intra-chromosome epistasis effects mostly in the 80–92 Mb region (Figure 7g), with peak effects between *rs136961414* in *LOC100138004* and *rs134391498* in *SLC4A4* Figure 7h). Similar to milk and protein yields, *rs109452259* between *GC* and *NPFFR2* and five SNPs in *SLC4A4* had the #3 and #8–11 effects of the chromosome.

Chr26 had scattered intra-chromosome epistasis effects in the 12–32 Mb region (Figure 7i) with peak effect between *rs42092371* in *NEURL1* and *rs41604819* in *SORCS1* (Figure 7j).

### 3.7. Inter-Chromosome Epistasis Effects of Fat Percentages

**Figure 8:**
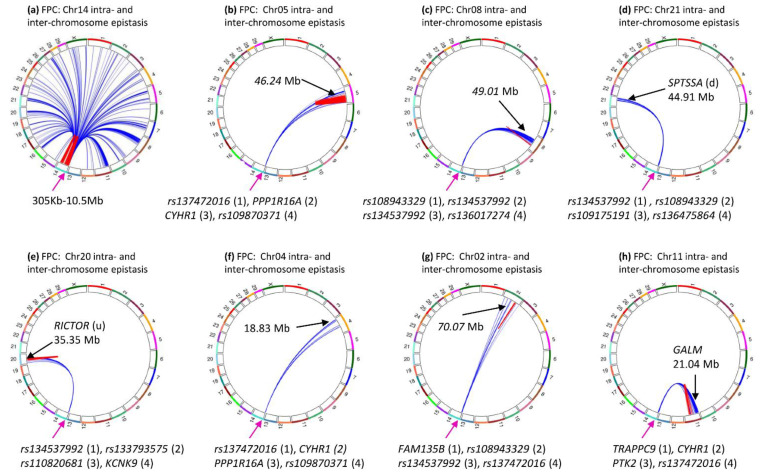
Examples of inter-chromosome epistasis effects between the same Chr14 region and different chromosomes for fat percentage (log10(1/p) ≥ 12.82). Each number on the outer circle is the chromosome number, red color represents intra-chromosome epistasis effects, and blue color represents inter-chromosome epistasis effects. Number in () is the effect ranking. The arrow indicates the Chr14 region that interacted with every chromosome for fat percentage. FPC = fat percentage. (**a**): Inter-chromosome epistasis effects between 26 chromosomes and the 0.3-10.5 Mb region of Chr14 for fat percentage. (**b**): Inter-chromosome epistasis effects between Chr05 and the 0.3-10.5 Mb region of Chr14 for fat percentage. (**c**): Inter-chromosome epistasis effects between Chr08 and the 0.3-10.5 Mb region of Chr14 for fat percentage. (**d**): Inter-chromosome epistasis effects between Chr21 and the 0.3-10.5 Mb region of Chr14 for fat percentage. (**e**): Inter-chromosome epistasis effects between Chr20 and the 0.3-10.5 Mb region of Chr14 for fat percentage. (**f**): Inter-chromosome epistasis effects between Chr04 and the 0.3-10.5 Mb region of Chr14 for fat percentage. (**g**): Inter-chromosome epistasis effects between Chr02 and the 0.3-10.5 Mb region of Chr14 for fat percentage. (**h**): Inter-chromosome epistasis effects between Chr11 and the 0.3-10.5 Mb region of Chr14 for fat percentage.

The most surprising inter-chromosome epistasis effects were those for fat percentage: all 29 chromosomes interacted with a Chr14 region with *DGAT1*, which had the most significant SNP effect for fat percentage [19]. Figure 2a shows 27 of the 30 chromosomes had significant inter-chromosome epistasis effects for fat percentage (log10(1/p) ≥ 12.82) among the top 50,000 pairs. However, 26 of the 27 chromosomes each only interacted with the same 0.3–10.5 Mb region of Chr14 (Figure 8a) with A × A effects. Only one inter-chromosome effect did not involve the Chr14 region: the D × D effect between Chr22 and Chr23 (Figure S2, Table S2). These results were from the 80 K SNP set that had more intra-chromosome epistasis effects (98.1%, Table 1) and fewer inter-chromosome epistasis effects (1.9%, Table 1) than those from the 60 K SNP set that had 94% intra- and 6% inter-chromosome epistasis effects. From the 60 K, all 29 chromosomes interacted with the same Chr14 region (Figure S2, Table S2). Figure 8b–h shows some 80 K examples showing each chromosome specifically interacted with the 0.3–10.5 Mb region of chr14. Such a high degree of specificity of the 0.3–10.5 Mb region of chr14 for interactions with other chromosomes provided evidence implicating a potential global regulatory role of this region for fat percentage and indicated that fat percentage likely had genetic contributions from the entire genome.

The analysis of the 0.3–10.5 Mb region of Chr14 showed that two regions flanking *DGAT1* had the most significant and frequent inter-chromosome epistasis effects, the *PPP1R16A-CYHR1* region at 0.46–0.50 Mb about 120 Kb upstream of *DGAT1* and the 2.33–3.34 Mb intergenic region about 1.71 Mb downstream of *DGAT1* including *rs134537992* at 242.1119 Kb with the most significant inter-chromosome epistasis effects (Figure 2b). Although *DGAT1* was only 0.12 Mb from the *PPP1R16A-CYHR1* region that had over 200 inter-chromosome epistasis effects, *DGAT1* was not involved in 98.75% of the 954 pairs of inter-chromosome epistasis effects, with a total of 12 (1.25%) effects and the highest effect ranking of #332 for inter-chromosome epistasis effects. In contrast, *TRAPPC9* had 73 (7.65%) of the 954 inter-chromosome epistasis effects and the #2 effect. The approximately 100 Kb intergenic region of 2.33–2.42 Mb had six SNPs and five of these six SNPs had 159 significant inter-chromosome epistasis effects with 19 chromosomes (chromosomes 1, 2, 7–9, 11, 12, 15, 17–24, 26, 28, X), including the most significant effect of *rs134537992*. The 0.3–10.5 Mb region had a gap of inter-chromosome epistasis effects at 87.65 Kb–2.33 Mb (Figure 2b), a 2.24 Mb gap without inter-chromosome epistasis effects but with many intra-chromosome epistasis effects (Figure 3a, Table S1), many SNP effects [19], and at least 44 coding genes.

Chr05 had 63 SNPs in the 0.13–119.07 Mb region interacting with the 0.46–5.68 Mb region of Chr14, showing that virtually the entire Chr05 of 120.09 Mb interacted with the same Chr14 region only, noting that nearly the entire Chr05 also had intra-chromosome epistasis effects for milk, fat and protein yields. Chr11 had the second largest number of SNPs interacting with the Chr14 regions, with 259 SNPs in the 0.65–62.52 Mb region interacting with the 0.46–6.25 Mb region of Chr14. Chr08 had the second largest number of SNPs interacting with the Chr14 regions, with 137 SNPs in the 32.72–81.71 Mb region interacting with the 0.46–6.76 Mb region of Chr14. It is interesting to note that both SNPs in *DGAT1* had significant inter-chromosome epistasis effects with *rs109477201* of Chr05, although *DGAT1* only had 1.25% of all inter-chromosome epistasis effects.

### 3.8. Inter-Chromosome Epistasis Effects of Protein Percentage and Yield Traits

**Figure 9:**
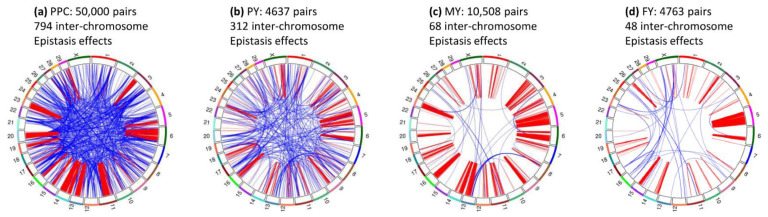
Inter-chromosome epistasis effects with log10(1/p) ≥ 12 for protein percentage and three yield traits. Each number on the outer circle is the chromosome number, red color represents intra-chromosome epistasis effects, and blue color represents inter-chromosome epistasis effects. PPC = protein percentage. PY = protein yield. MY = milk yield. FY = fat yield. (**a**): Intra- and inter-chromosome epistasis effects of protein percentage. (**b**): Intra- and inter-chromosome epistasis effects of protein yield. (**c**): Intra- and inter-chromosome epistasis effects of milk yield. (**d**): Intra- and inter-chromosome epistasis effects of fat yield.

The inter-chromosome epistasis effects of protein percentage and the three yield traits involved many chromosome pairs (Figure 9) but did not have the specificity observed for inter-chromosome epistasis effects of Chr14 for fat percentage (Figure 8).

**Figure 10:**
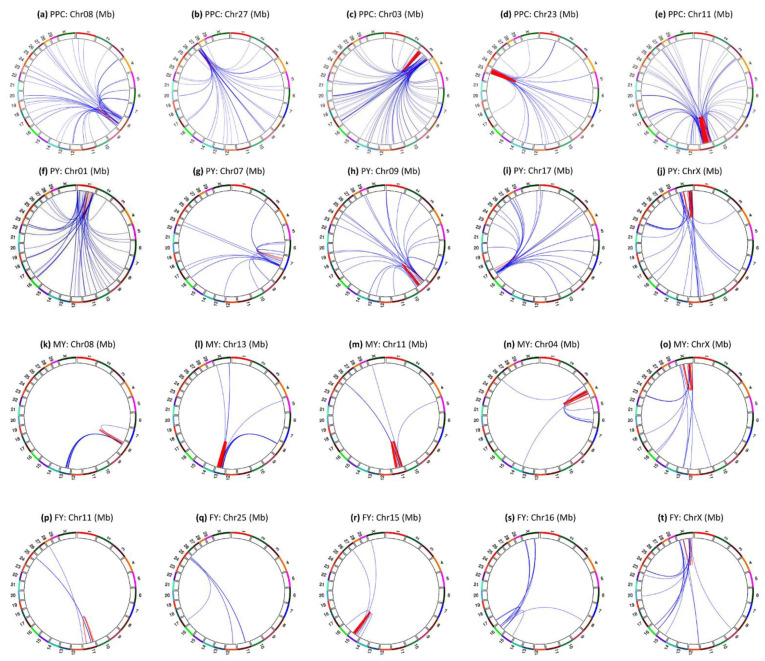
Examples of inter-chromosome epistasis effects with log10(1/p) ≥ 12 for protein percentage and the three yield traits. Each number on the outer circle is the chromosome number, red color represents intra-chromosome epistasis effects, and blue color represents inter-chromosome epistasis effects. PPC = protein percentage. PY = protein yield. MY = milk yield. FY = fat yield. (**a**–**e**): Examples of inter-chromosome epistasis effects for protein percentage. (**f**–**j**): Examples of inter-chromosome epistasis effects for protein yield. (**k**–**o**): Examples of inter-chromosome epistasis effects for milk yield. (**p**–**t**): Examples of inter-chromosome epistasis effects for fat yield.

Protein percentage had 794 inter-chromosome epistasis effects, fewer than the 954 inter-chromosome epistasis effects for fat percentage (Table 1), and every chromosome interacted with most of the other chromosomes (Figure 9a and Figure S2), unlike fat percentage where every chromosome virtually interacted with the same Chr14 region only (Figure 8a and Figure S2). Figure 10a–e show examples where every chromosome that interacted with most of the other chromosomes for protein percentage.

Protein yield (Figure 9b) had 312 significant inter-chromosome epistasis effects with similar patterns as protein percentage, i.e., every chromosome had inter-chromosome epistasis effects with some other chromosomes. Figure 10f–j show examples of inter-chromosome epistasis effects of protein yield.

Milk yield had 68 (0.6%) inter-chromosome epistasis effects involving chromosome pairs between 20 chromosomes (Figure 9c). Half of these 68 effects were between Chr08 and Chr13, with 29 effects between 5 SNPs in *LOC101904827* on Chr08 and 14 SNPs in the 20.63–46.24 Mb region of Chr13. Figure 10k–o show examples of inter-chromosome epistasis effects of milk yield.

Fat yield had 48 (1.0%) inter-chromosome epistasis effects involving chromosome pairs between 22 chromosomes (Figure 9d). A surprise was that Chr05 with intra-chromosome epistasis effects virtually covering the entire chromosome (Figure 7c) did not have significant inter-chromosome epistasis effects. Figure 10p–t show examples of inter-chromosome epistasis effects of fat yield.

### 3.9. Epistasis Effects of Daughter Pregnancy Rate

**Figure 11:**
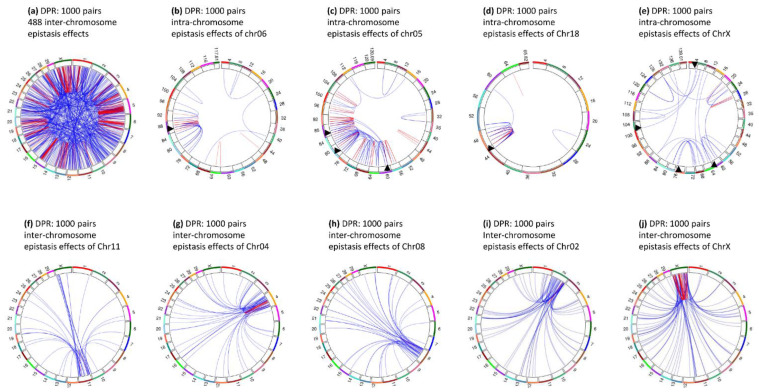
Intra- and inter-chromosome epistasis effects of daughter pregnancy rate (top 1000 pairs). For intra-chromosome epistasis effects, each number on the outer circle is the chromosome number, red color indicates intra-chromosome epistasis effects, and blue color indicates inter-chromosome epistasis effects. For inter-chromosome epistasis effects, each number on the outer circle is the chromosome distance, red color indicating intra-4 Mb epistasis effects and blue color indicating inter-4 Mb epistasis effects. DPR = daughter pregnancy rate. (**a**): Intra- and inter-chromosome epistasis effects of all chromosomes for daughter pregnancy rate. (**b**): Intra-chromosome epistasis effects of Chr06 for daughter pregnancy rate. (**c**): Intra-chromosome epistasis effects of Chr05 for daughter pregnancy rate. (**d**): Intra-chromosome epistasis effects of Chr18 for daughter pregnancy rate. (**e**): Intra-chromosome epistasis effects of ChrX for daughter pregnancy rate. (**f**): Inter-chromosome epistasis effects of Chr11 for daughter pregnancy rate. (**g**): Inter-chromosome epistasis effects of Chr04 for daughter pregnancy rate. (**h**): Inter-chromosome epistasis effects of Chr08 for daughter pregnancy rate. (**i**): Inter-chromosome epistasis effects of Chr02 for daughter pregnancy rate. (**j**): Inter-chromosome epistasis effects of ChrX for daughter pregnancy rate.

Daughter pregnancy rate only had one significant epistasis effect with log10(1/p) ≥ 12 (Figure 1f), and this epistasis effect was an intra-chromosome A × A effect between *rs133222820* in *TMPRSS11E* and rs43470988 (*RUFY3-GRSF1*) of Chr06. Although other effects did not reach the Bonferroni significance, several epistasis effects involved genes with reported fertility effects. These included *EIF5B* (inter-chromosome epistasis effect between *rs43652424* in *EIF5B* of Chr11 and *rs136798451* flanked by *RAP2C* and *MBNL3* of ChrX) associated with female sterility [34], *RAP2C* (inter-chromosome epistasis effect) associated with gestational duration and/or spontaneous preterm birth [35], *PKP2* (intra-chromosome epistasis effect of Chr05) associated with cow conception rate [36], *HSD17B6* (intra-chromosome epistasis effect of Chr05) associated with polycystic ovary syndrome [37], and *SOX5* (intra-chromosome epistasis effect of Chr05) associated with nonobstructive azoospermia susceptibility [38]. Figure 11a shows the top 1000 epistasis effects for daughter pregnancy rate, with 55.2% intra-chromosome and 48.8 inter-chromosome epistasis effects. Figure 11b–e show examples of intra-chromosome and Figure 11f–j shows examples of inter-chromosome epistasis effects.

## 4. Discussion

### 4.1. Complex Epistasis Effects Existed in U.S. Holstein Cattle

The epistasis results of the five milk production traits showed that complex epistasis effects existed in U.S. Holstein cattle. Every production trait had intra-chromosome epistasis effects covering large chromosome distances, and inter-chromosome epistasis effects existed. The Chr05 example for fat yield was the largest chromosome segment (nearly the entire chromosome) covered by intra-chromosome epistasis effects. In addition, inter-chromosome epistasis effects also existed involving most chromosomes for every trait. For fat percentage, a Chr14 region had inter-chromosome epistasis effects with all other chromosomes. The complexity of the epistasis effects in this study was unprecedented.

### 4.2. Genetic Selection Based on Genome-Wide SNP Additive Effects Likely Accounted for Most Intra-Chromosome A × A Effects

The chromosome regions with highly significant intra-chromosome A × A epistasis effects of the five production traits overlapped the chromosome regions with highly significant SNP additive effects we previously reported from the same data set. Given that genomic prediction using single-SNP additive models for the production traits was successful, we hypothesize that genome-wide SNP additive effects likely accounted for most intra-chromosome A × A effects. The linkage between SNPs with intra-chromosome A × A effects should have contributed to the success of genomic prediction using SNP additive models for the production traits. As the percentage of inter-chromosome epistasis effects increases, the effectiveness of SNP prediction models is expected to decrease, as discussed next. Based on this assumption, SNP prediction models should be most effective for fat and protein percentages that had the highest frequencies of intra-chromosome A × A effects, followed by milk yield, fat yield and protein yield. Statistical significance is another factor affecting the contribution of intra- or inter-chromosome epistasis effects to the trait. For the five production traits, intra-chromosome epistasis effects were more significant than inter-chromosome effects, e.g., log10(1/p) was in the range of 13–537 for intra- and 13–23 for inter-chromosome epistasis effects of fat percentage (Table 1), and the other four production traits had a similar trend. Daughter pregnancy rate was different with similar statistical significance for both intra- and inter-chromosome epistasis effects, log10(1/p) was in the range of 6–13 for intra- and 6–11 for inter-chromosome epistasis effects. Combining the frequency and statistical significance of intra-chromosome epistasis effects, genomic selection using SNP additive models is expected to be highly effective for the five production traits.

### 4.3. Inter-Chromosome Epistasis Effects Could Be a Genetic Mechanism for Lack of Selection Response and Low Heritability

Daughter pregnancy rate had the highest frequency of inter-chromosome epistasis effects (84.2%, Table 1), and more than half (52.9%) of the top 50,000 epistasis effects were A × A effects. Although only one epistasis effect reached the Bonferroni significance, the epistasis results provided the first indications that inter-chromosome A × A effects may be the primary genetic effects of daughter pregnancy rate. Assuming this was true, inter-chromosome A × A effects would be consistent with the fact that genetic selection for daughter pregnancy rate was less responsive than the yield traits because genetic selection for many independent variants would be more difficult than selection for many lined variants. As discussed later, we hypothesize that marginal effect of many epistasis effects of linked loci is a contributing factor to highly significant SNP effect. However, such marginal effect is unavailable for inter-chromosome epistasis effects of independent SNPs on different chromosomes.

### 4.4. Chr14-Specific Inter-Chromosome A × A Epistasis Effects Increase the Statistical Confidence of the Epistasis Results

Although the statistical significance for declaring significant epistasis effects was the stringent significant level of 5% genome-wide false positives with the Bonferroni multiple testing correction, large numbers of epistasis effects for the five production traits reached this Bonferroni significance, log10(1/p) ≥ 12, e.g., all the top 50,000 epistasis effects of fat or protein percentage were significant. This raised the question whether false positive effects were substantially more than the expected 5% genome-wide false positives. While a definitive answer to this question was unavailable, the inter-chromosome A × A epistasis effects between the 0.3–10.5 Mb Chr14 region and all chromosomes for fat percentage should increase the statistical confidence of the epistasis results, because the high degree of specificity of all 29 chromosomes interacting with the same chromosome region could not happen by chance.

### 4.5. An Intergenic Variant May Have an Important Role for Inter-Chromosome Epistasis Effects of Fat Percentage

The 0.9 Mb intergenic region at 2.33–2.42 Mb of Chr14 had six SNPs and five of these SNPs had significant inter-chromosome epistasis effects including the most significant effect of *rs134537992* at 242.1119 Kb for fat percentage. This 0.9 Mb intergenic region was 113.637 Kb from *LOC101901918* or 311.437 Kb from *TSNARE1* upstream, and 88.851 Kb from *SLC45A4* downstream. These three flanking genes had eight SNPs, one in *LOC101901918*, five in *TSNARE1* and two in *SLC45A4*, but none of these eight SNPs had a significant inter-chromosome effect. These results would be consistent with the hypothesis that the 0.9 Mb intergenic region at 2.33–2.42 Mb had its own biological functions affecting many chromosomes for fat percentage rather than linked effects due to linkage disequilibrium with adjacent coding genes. The 2.24 Mb gap in inter-chromosome epistasis effects at 87.65 Kb–2.30 Mb should further support this hypothesis. This gap region had many intra-chromosome epistasis effects (Figure 3a, Table S1), many SNP effects [19], and at least 44 coding genes, but did not have significant inter-chromosome epistasis effects. Given that this 2.24 Mb gap region at 87.65 Kb–2.30 Mb was so close to the *PPP1R16A-CYHR1* region at 0.46–0.50 Mb and the 2.33–3.34 Mb intergenic region with significant inter-chromosome epistasis effects, the coding genes in this 2.24 Mb gap region did not have biological functions for significant inter-chromosome epistasis effects. These results indicated that the 0.9 Mb segment of noncoding DNA at 2.33–3.34 Mb harboring *rs134537992* had potential regulatory functions affecting many chromosomes for fat percentage.

### 4.6. Causal or Linked Epistasis Effects

The results in this study did not provide evidence about potential causal variants with epistasis effects, but had indications about potential involvement of linkage disequilibrium in clusters of epistasis effects of the production traits, and an approximate chromosome region with causal effects for inter-chromosome epistasis effects. Fat percentage about is an ideal trait to discuss the issue of causal and linked effects due to the unique statistical significance and patterns of the epistasis effects. The chromosome regions with large clusters of intra-chromosome epistasis effects mostly contained the chromosome regions with large clusters of SNP effects including the Chr14 region with intra-chromosome epistasis effects. We previously reported that the region containing *DGAT1* about 6.79 Mb in size had strong linkage disequilibrium, and the removal of the *DGAT1* effect from the phenotypic values eliminated 63% of the top 1% effects in the region, showing that the top 1% SNP effects in that 6.79 Mb region involved both causal and linked effects. Since A × A epistasis effect of a SNP pair in this study was the contrast of the four haplotype average values and each haplotype frequency was affected by linkage disequilibrium between the two SNPs [30], A × A intra-chromosome epistasis effects due to linkage should have existed and contributed to the large clusters of intra-chromosome epistasis effects for the five production traits. Inter-chromosome epistasis effects are expected to be unaffected by linkage disequilibrium between the two SNPs of each SNP pair but some effects still could have contributions from linkage disequilibrium between a SNP adjacent to another SNP that had a significant inter-chromosome epistasis effect. An example of this possibility is the 0.9 Mb intergenic region at 2.33–2.42 Mb of Chr14 where five of the six SNPs had significant inter-chromosome epistasis effects including the most significant effect of *rs134537992* at 242.1119 Kb for fat percentage. Some of these five significant epistasis effects could be due to linkage to a causal effect nearby. However, inter-chromosome epistasis effects clearly were less affected by linkage, e.g., the 2.24 Mb gap region at 87.65 Kb–2.30 Mb without significant inter-chromosome epistasis effects was very close to the *PPP1R16A-CYHR1* region at 0.46–0.50 Mb and the 2.33–3.34 Mb intergenic region with significant inter-chromosome epistasis effects.

## 5. Conclusions

The epistasis results showed that complex intra- and inter-chromosome epistasis effects existed in U.S. Holstein cattle. Intra-chromosome A × A effects were the main epistasis effects for the five production traits, whereas inter-chromosome A × A effects were the main epistasis effects for daughter pregnancy rate. Chromosome regions with highly significant intra-chromosome epitasis effects mostly overlapped the chromosome regions with highly significant SNP effects we previously reported using the same data. A chr14 region widely reported to have highly significant SNP effects for fat percentage interacted with all other chromosomes, and this result provided evidence for a potential global regulatory role of this Chr14 region.

## Figures and Tables

**Table 1:** Frequencies and statistical significance of intra- and inter-chromosome pairwise epistasis effects among the top 50,000 epistasis effects.

	Intra-Chromosome Epistasis (% of Intra-Chromosome SNP Pairs: 3.6)			Inter-Chromosome Epistasis (% of Inter-Chromosome SNP Pairs: 96.4)		
	Freq (%)	Log10(1/p)	Pairs with log10(1/p) ≥ 12	Freq (%)	Log10(1/p)	Pairs with log10(1/p) ≥ 12
FPC	98.1	13–537	49,046 (98.1%)	1.9	13–23	954 (1.9%)
PPC	98.4	17–211	49,206 (98.4%)	1.6	17–82	794 (1.6%)
MY	89.4	9–97	10,440 (20.9%)	10.6	13–19	68 (0.6%)
FY	70.1	7–40	4715 (9.4%)	29.9	7–14	48 (1.0%)
PY	61.7	7–51	4325 (8.7%)	39.3	7–21	312 (6.7%)
DPR	15.8	6–13	1 (0.002%)	84.2	6–11	0

Freq = frequency. FPC = fat percentage. PPC = protein percentage. MY = milk yield. FY = fat yield. PY = protein yield.

**Table 2:** Frequencies of A × A, A × D/D × A and D × D epistasis effects within intra- and inter-chromosome pairwise epistasis effects among the top 50,000 epistasis effects.

		Intra-Chromosome Epistasis			Inter-Chromosome Epistasis		
		A × A	A × D and D × A	D × D	A × A	A × D and D × A	D × D
FPC	Count	47,752	1023	271	952	0	1
	%	95.5	2.1	0.5	1.9	0.0	0.0
PPC	Count	45,379	2936	1116	10	3	780
	%	90.8	5.9	2.2	0.0	0.0	1.6
MY	Count	36,387	5978	2355	5197	35	48
	%	72.8	12.0	4.7	10.4	0.1	0.1
FY	Count	30,225	3767	1401	14,317	188	92
	%	60.5	7.5	2.8	28.6	0.4	0.2
PY	Count	20,582	7461	2840	17,468	200	1448
	%	41.2	14.9	5.7	34.9	0.4	2.9
DPR	Count	7122	602	196	26,430	10,531	5118
	%	14.2	1.2	0.4	52.9	21.1	10.2

FPC = fat percentage. PPC = protein percentage. MY = milk yield. FY = fat yield. PY = protein yield.

## Data Availability

The original genotype data are owned by third parties and maintained by the Council on Dairy Cattle Breeding (CDCB). A request to CDCB is necessary for getting data access on research, which may be sent to: João Dürr, CDCB Chief Executive Officer (joao.durr@cdcb.us). All other relevant data are available in the manuscript, Supplementary Materials.

## Supplementary Materials

The following are available online at https://www.mdpi.com/article/10.3390/genes12071089/s1, Figure S1: Intra-chromosome epistasis effects with log(1/p) ≥ 12, Figure S2: Inter-chromosome epistasis effects with log(1/p) ≥ 12, Table S1: Significant intra-chromosome epistasis effects, Table S2: Significant inter-chromosome epistasis effects.
