# Supplementary material for: A Large-Scale Genome-Wide Association Study of Epistasis Effects of Production Traits and Daughter Pregnancy Rate in U.S. Holstein Cattle
Source: Genes (Basel). 2021 Jul 18;12(7):1089. doi: 10.3390/genes12071089 (PMC8304971; doi:10.3390/genes12071089)
Supplement: Supplementary file 1 [file genes-12-01089-s001.zip › supplementary/Figure_S1.pdf]

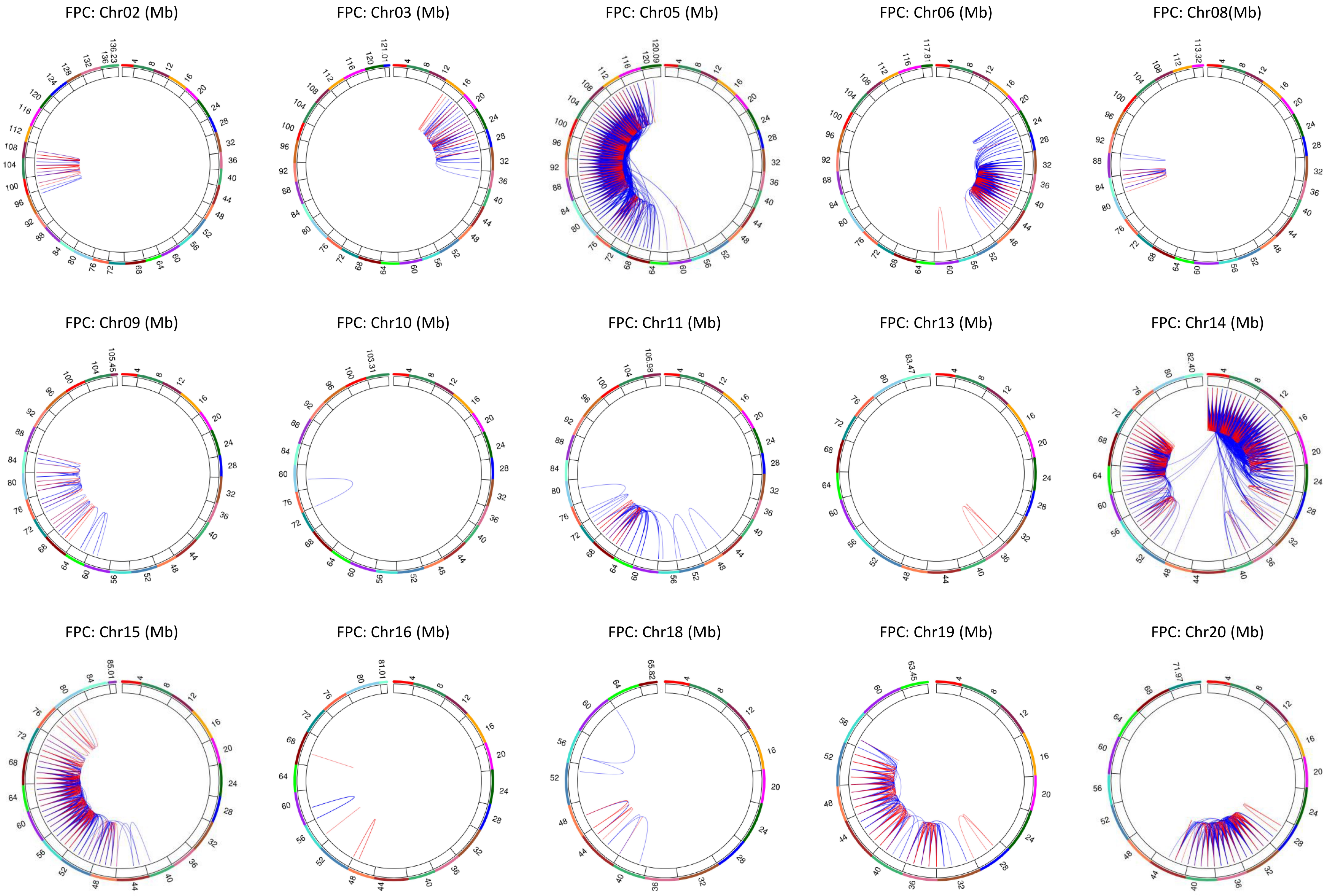
FPC: Chr02 (Mb)
FPC: Chr03 (Mb)
FPC: Chr05 (Mb)
FPC: Chr06 (Mb)
FPC: Chr08(Mb)
FPC: Chr09 (Mb)
FPC: Chr10 (Mb)
FPC: Chr11 (Mb)
FPC: Chr13 (Mb)
FPC: Chr14 (Mb)
FPC: Chr15 (Mb)
FPC: Chr16 (Mb)
FPC: Chr18 (Mb)
FPC: Chr19 (Mb)
FPC: Chr20 (Mb)

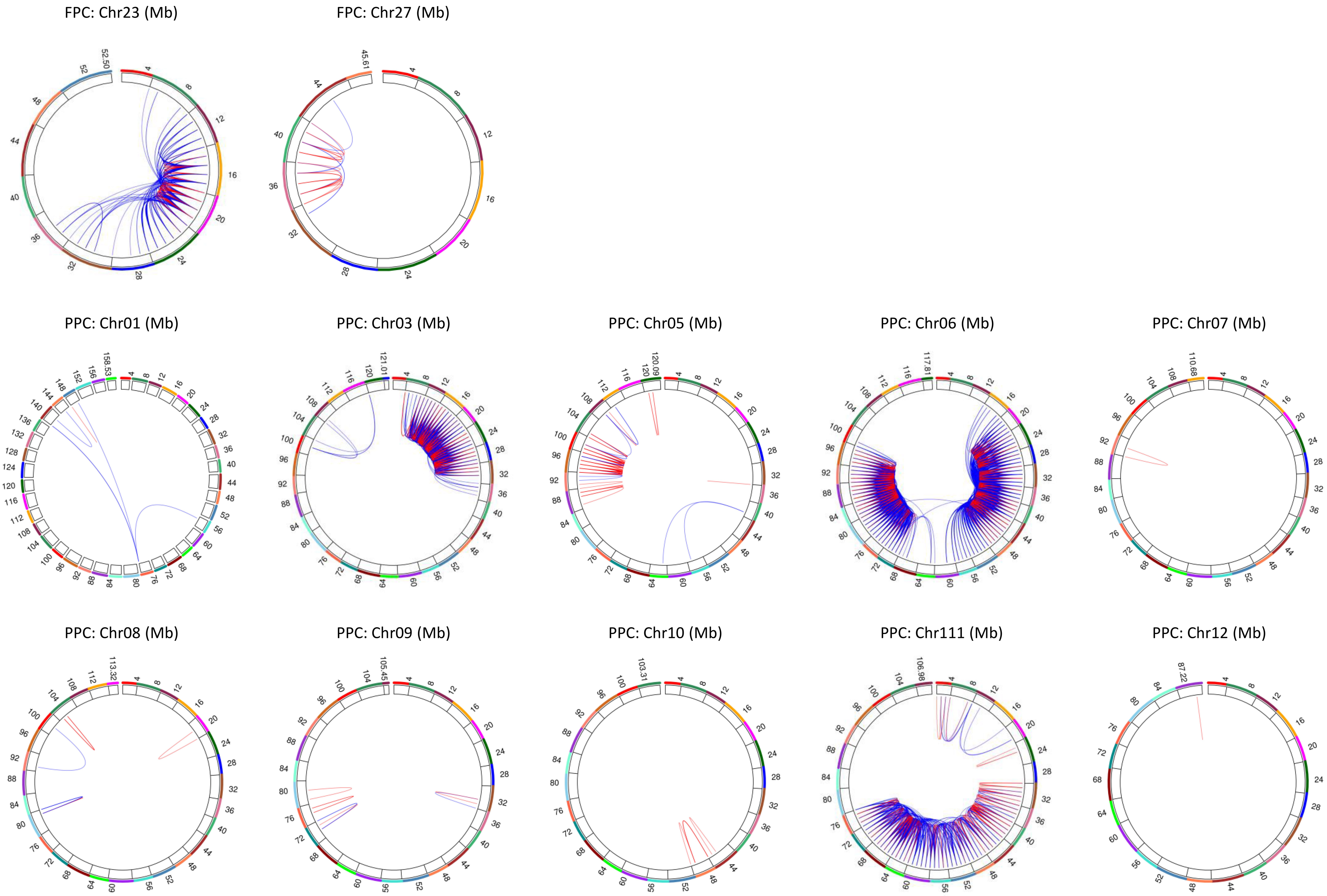
FPC: Chr23 (Mb)
FPC: Chr27 (Mb)
PPC: Chr01 (Mb)
PPC: Chr03 (Mb)
PPC: Chr05 (Mb)
PPC: Chr06 (Mb)
PPC: Chr07 (Mb)
PPC: Chr08 (Mb)
PPC: Chr09 (Mb)
PPC: Chr10 (Mb)
PPC: Chr111 (Mb)
PPC: Chr12 (Mb)

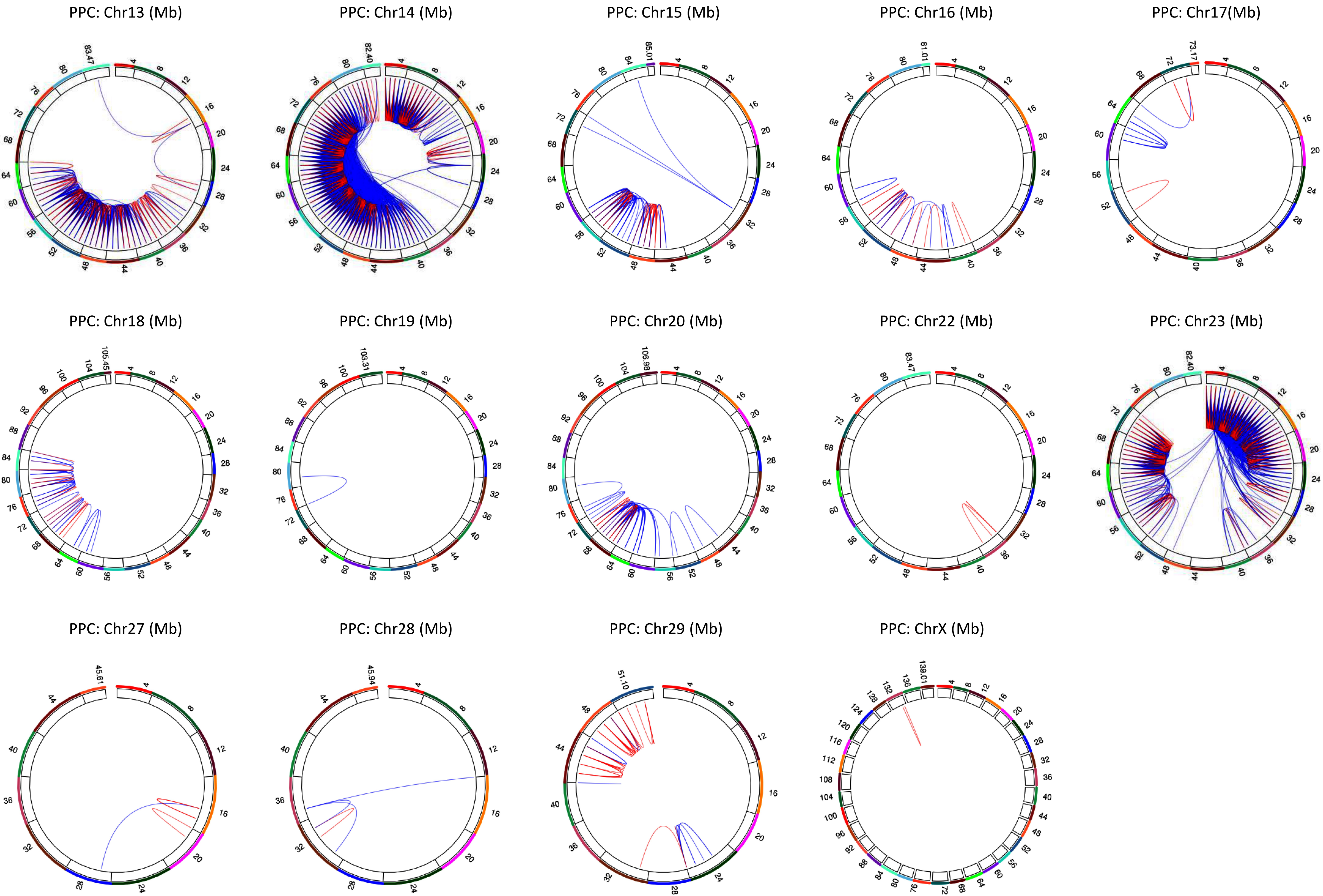
PPC: Chr13 (Mb)
PPC: Chr14 (Mb)
PPC: Chr15 (Mb)
PPC: Chr16 (Mb)
PPC: Chr17(Mb)
PPC: Chr18 (Mb)
PPC: Chr19 (Mb)
PPC: Chr20 (Mb)
PPC: Chr22 (Mb)
PPC: Chr23 (Mb)
PPC: Chr27 (Mb)
PPC: Chr28 (Mb)
PPC: Chr29 (Mb)
PPC: ChrX (Mb)

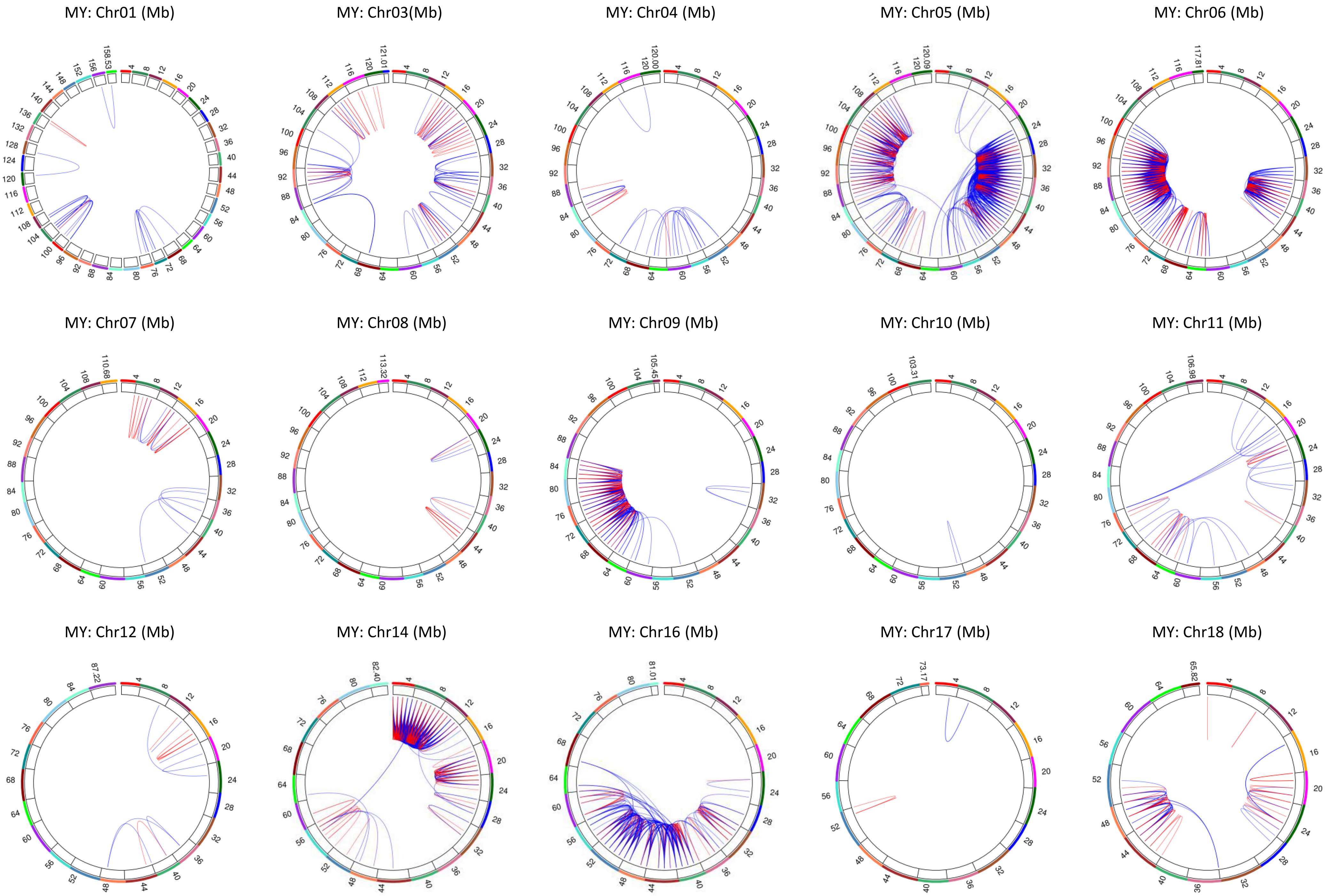
MY: Chr01 (Mb)
MY: Chr03(Mb)
MY: Chr04 (Mb)
MY: Chr05 (Mb)
MY: Chr06 (Mb)
MY: Chr07 (Mb)
MY: Chr08 (Mb)
MY: Chr09 (Mb)
MY: Chr10 (Mb)
MY: Chr11 (Mb)
MY: Chr12 (Mb)
MY: Chr14 (Mb)
MY: Chr16 (Mb)
MY: Chr17 (Mb)
MY: Chr18 (Mb)

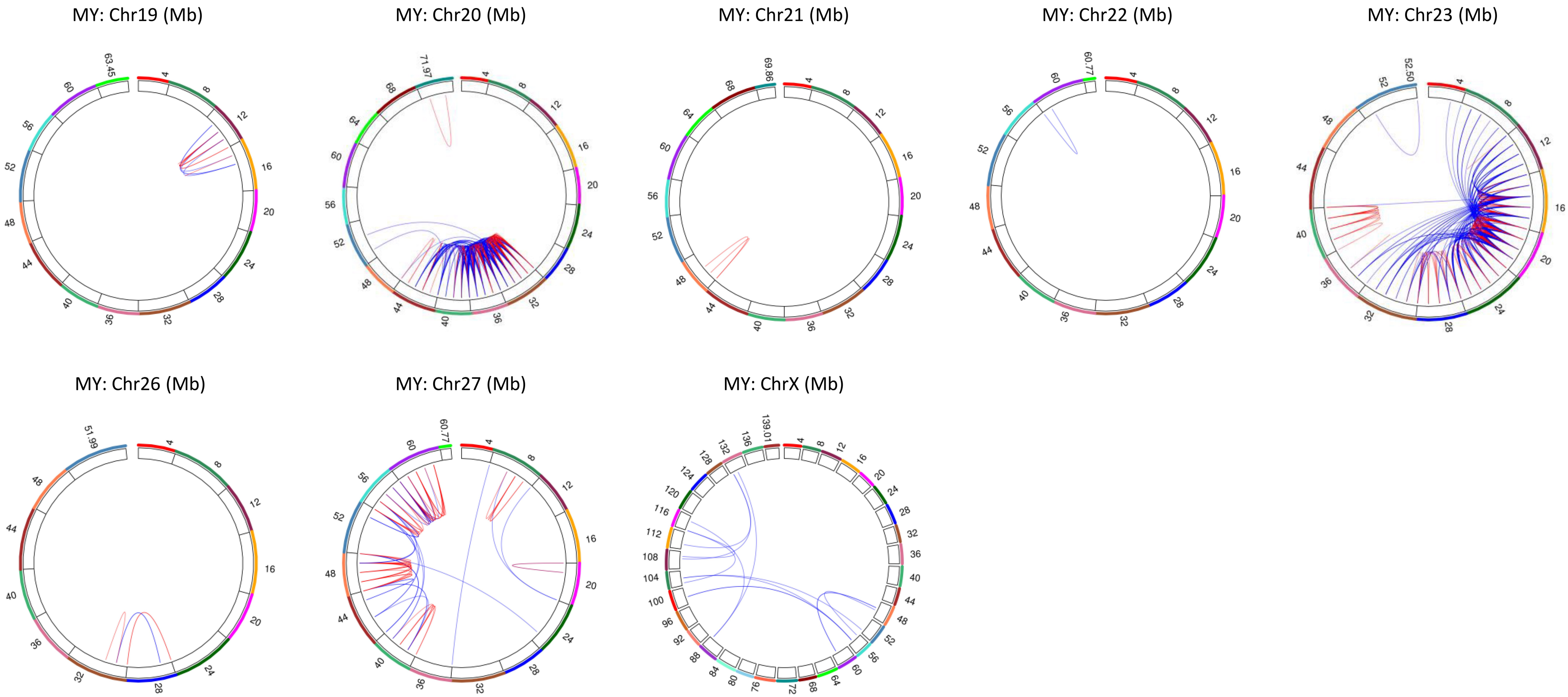
MY: Chr19 (Mb)
MY: Chr20 (Mb)
MY: Chr21 (Mb)
MY: Chr22 (Mb)
MY: Chr23 (Mb)
MY: Chr26 (Mb)
MY: Chr27 (Mb)
MY: ChrX (Mb)
63.45
71.97
69.86
60.77
52.50
51.99
60.77
139.01

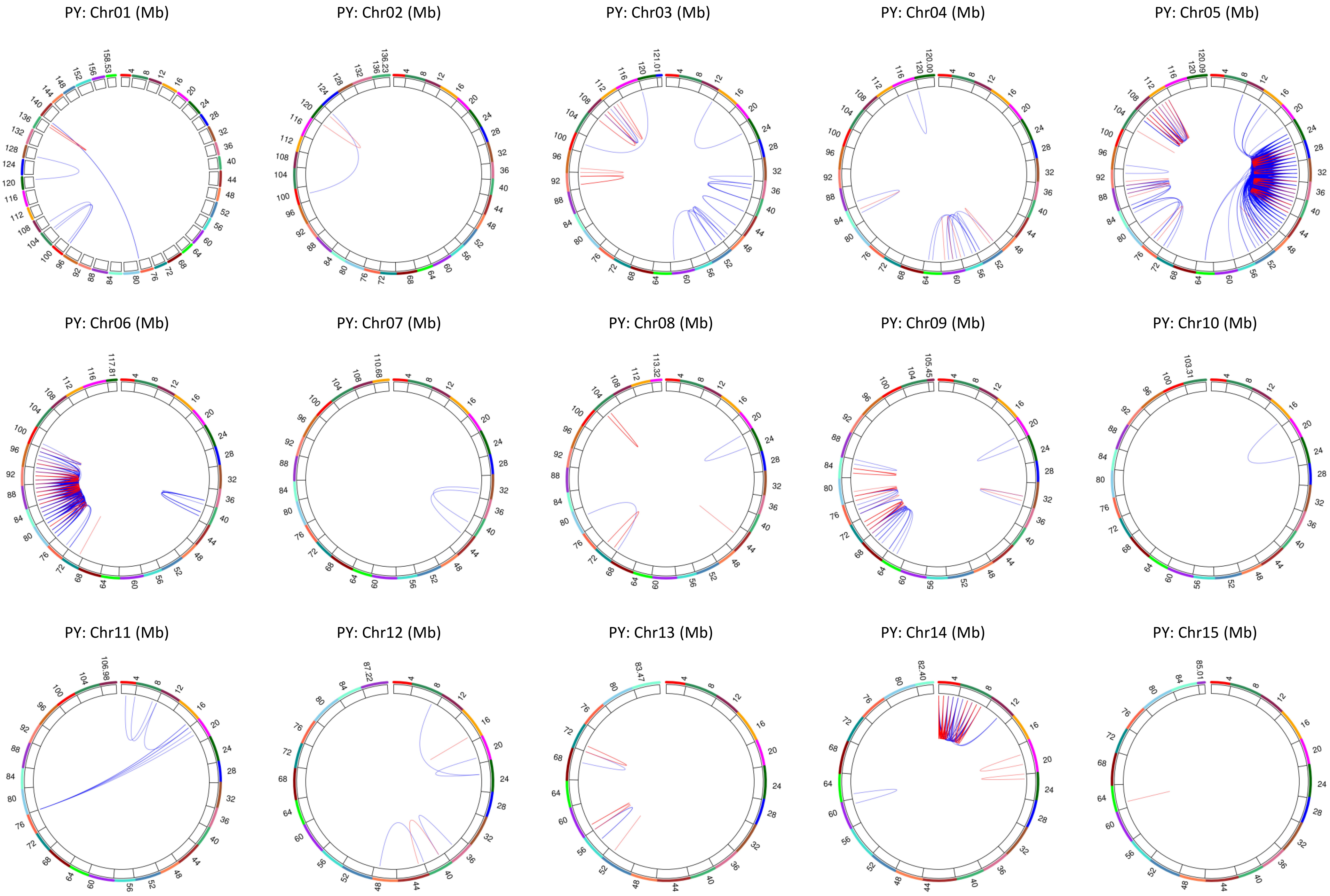
PY: Chr01 (Mb)
PY: Chr02 (Mb)
PY: Chr03 (Mb)
PY: Chr04 (Mb)
PY: Chr05 (Mb)
PY: Chr06 (Mb)
PY: Chr07 (Mb)
PY: Chr08 (Mb)
PY: Chr09 (Mb)
PY: Chr10 (Mb)
PY: Chr11 (Mb)
PY: Chr12 (Mb)
PY: Chr13 (Mb)
PY: Chr14 (Mb)
PY: Chr15 (Mb)

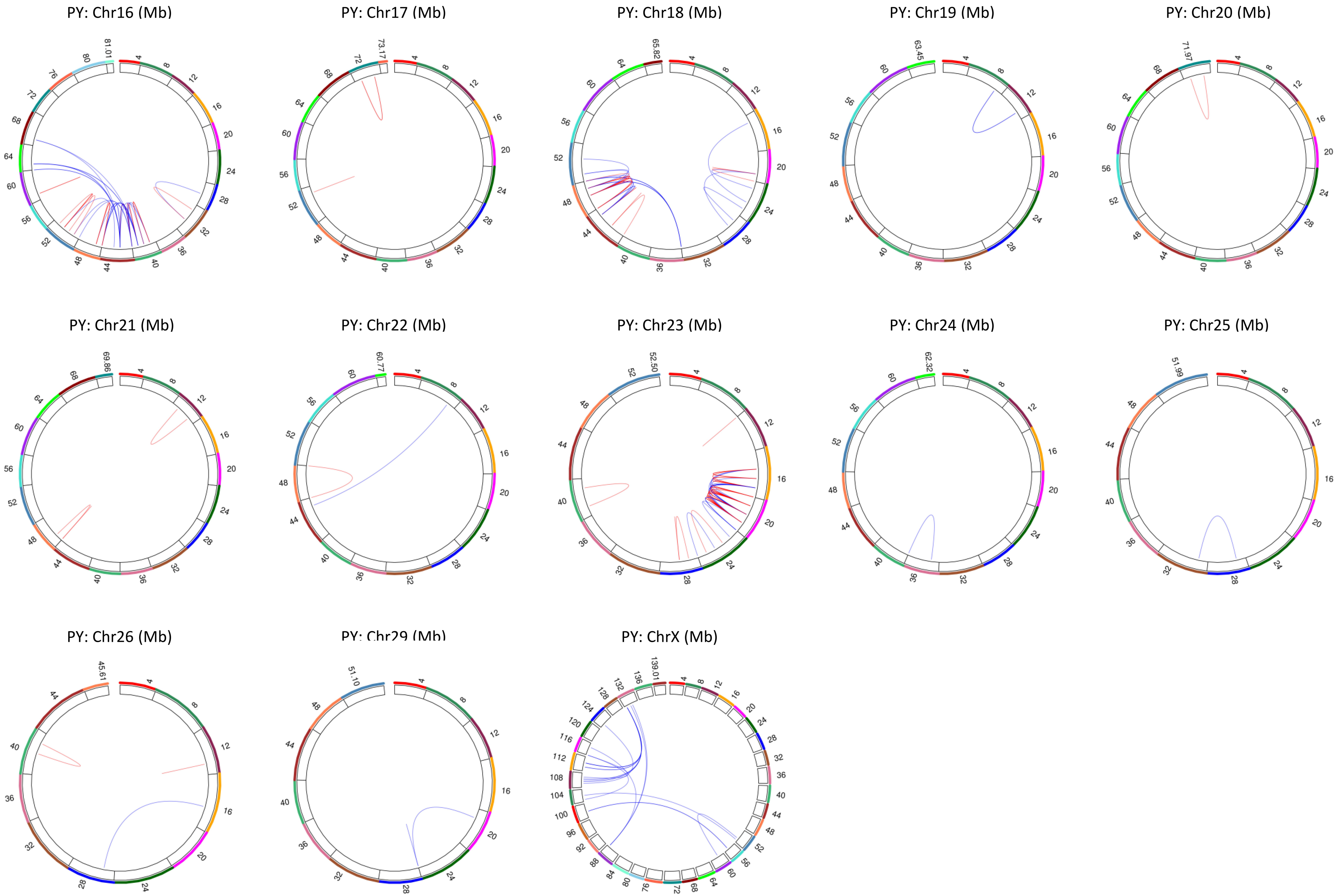
PY: Chr16 (Mb)
PY: Chr17 (Mb)
PY: Chr18 (Mb)
PY: Chr19 (Mb)
PY: Chr20 (Mb)
PY: Chr21 (Mb)
PY: Chr22 (Mb)
PY: Chr23 (Mb)
PY: Chr24 (Mb)
PY: Chr25 (Mb)
PY: Chr26 (Mb)
PY: Chr29 (Mb)
PY: ChrX (Mb)

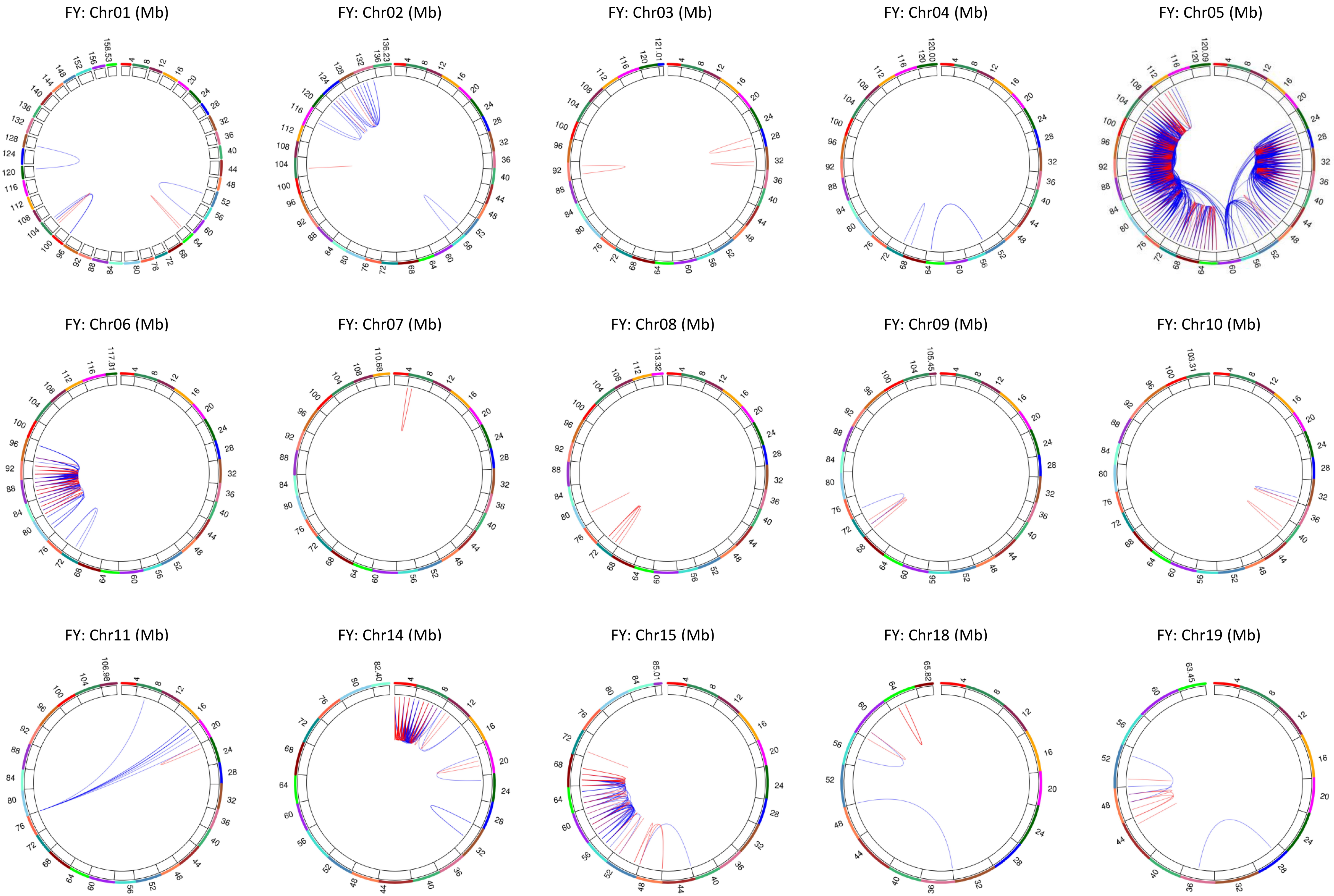
FY: Chr01 (Mb)
FY: Chr02 (Mb)
FY: Chr03 (Mb)
FY: Chr04 (Mb)
FY: Chr05 (Mb)
FY: Chr06 (Mb)
FY: Chr07 (Mb)
FY: Chr08 (Mb)
FY: Chr09 (Mb)
FY: Chr10 (Mb)
FY: Chr11 (Mb)
FY: Chr14 (Mb)
FY: Chr15 (Mb)
FY: Chr18 (Mb)
FY: Chr19 (Mb)

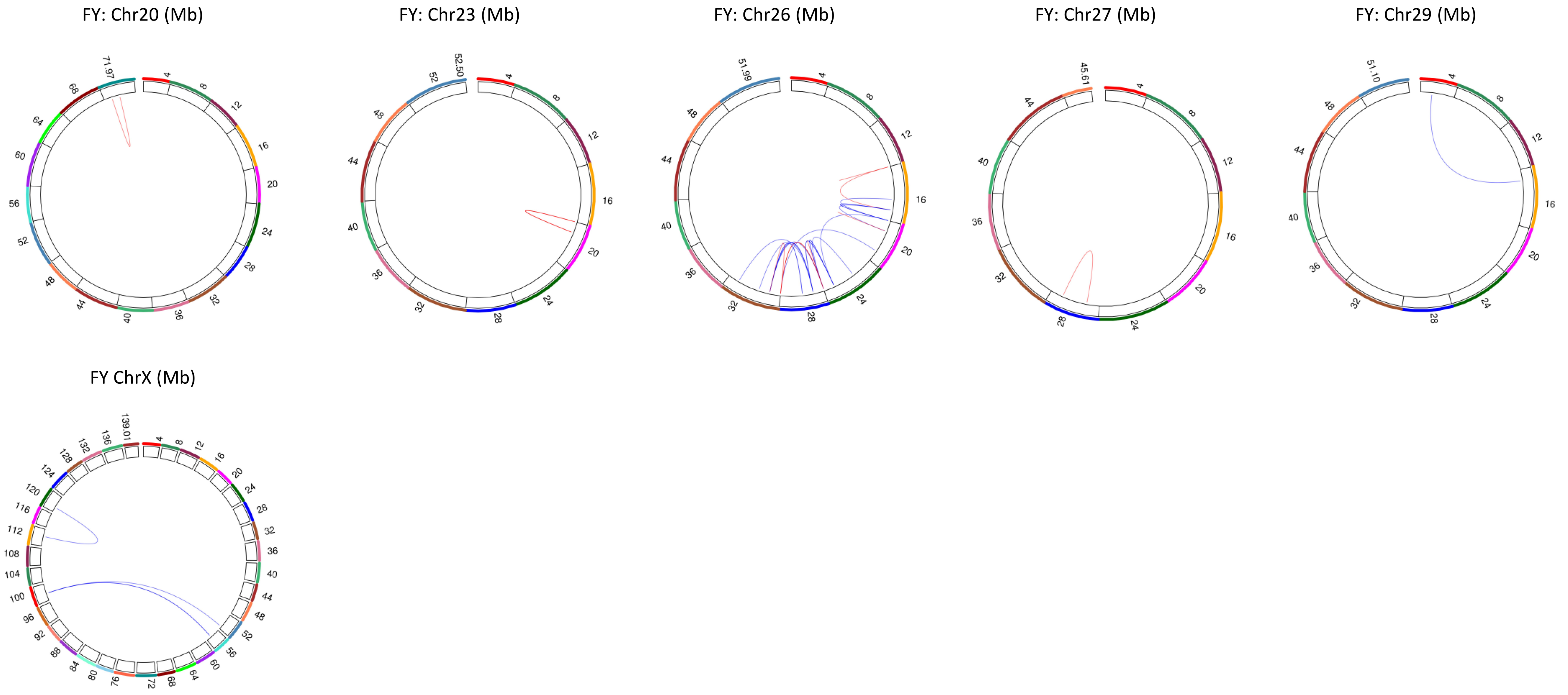

Figure S1. Intra-chromosome epistasis effects with log(1/p) ≥ 12. Each outer number is the chromosome distance in megabases (Mb), each chromosome is divided into 4-Mb segments, red color represents intra-4Mb or short-range epistasis effects, and blue color represents inter-4Mb or long-range epistasis effects. FPC = fat percentage, PPC = protein percentage, MY = milk yield, PY = protein yield, FY = fat yield.
